# Clinical Efficacy of Er,Cr:YSGG Laser for Deepithelialization of Free Gingival Grafts in Gingival Recession Treatment: A Randomized, Split-Mouth Clinical Trial

**DOI:** 10.3390/jcm14155335

**Published:** 2025-07-29

**Authors:** Artur Banyś, Jakub Fiegler-Rudol, Zuzanna Grzech-Leśniak, Rafał Wiench, Jacek Matys, Jamil A. Shibli, Kinga Grzech-Leśniak

**Affiliations:** 1 EMDOLA—European Master Degree in Oral Laser Application, Wroclaw Medical University, 50-367 Wroclaw, Poland; a.banys09@gmail.com; 2 Faculty of Medical Sciences in Zabrze, Medical University of Silesia, 40-055 Katowice, Poland; s88998@365.sum.edu.pl; 3 Department of Experimental Dentistry, Faculty of Medicine and Dentistry, Wroclaw Medical University, 50-367 Wroclaw, Poland; zuzanna.grzech-lesniak@student.umw.edu.pl; 4 Department of Periodontal Diseases and Oral Mucosa Diseases, Faculty of Medical Sciences in Zabrze, Medical University of Silesia, 40-055 Katowice, Poland; rwiench@sum.edu.pl; 5 Dental Surgery Department, Faculty of Medicine and Dentistry, Wroclaw Medical University, 50-367 Wroclaw, Poland; 6 Faculdade Israelita de Ciências da Saúde Albert Einstein, Hospital Israelita Albert Einstein, São Paulo 05652-900, Brazil; jashibli@ung.br; 7 Department of Periodontology, Dental Research Division, Guarulhos University, Guarulhos 07000-000, Brazil; 8 Department of Oral Medicine, Infection, and Immunity, Division of Periodontology, Harvard School of Dental Medicine, Boston, MA 02115, USA; 9 Laser Laboratory, Department of Integrated Dentistry, Faculty of Medicine and Dentistry, Wroclaw Medical University, 50-425 Wroclaw, Poland; 10 Department of Periodontics, School of Dentistry, Virginia Commonwealth University (VCA), Richmond, VA 23298, USA

**Keywords:** connective tissue graft, deepithelialized gingival graft, free gingival graft, laser deepithelialization, microsurgery, split-mouth study

## Abstract

**Bcakground**: The deepithelialized free gingival graft (DGG) technique provides high-quality connective tissue grafts (CTGs) with predictable outcomes for recession coverage. This study evaluates a novel method of free gingival graft (FGG) deepithelialization using an Er,Cr:YSGG laser (LDEE) for treating multiple gingival recessions. **Methods**: A split-mouth study was conducted on 46 (*n* = 46) recessions in nine patients (23 per test and control group). Sites were randomized. Full-thickness palatal grafts were harvested with a scalpel. In the test group (LDEE), deepithelialization was performed extraorally using an Er,Cr:YSGG laser (2780 nm; 2.5 W, 83.3 mJ, 30 Hz, 600 µm tip). In the control group (DEE), a 15c scalpel was used. All CTGs were applied using the modified coronally advanced tunnel (TUN) technique. Clinical parameters—recession depth (RD), keratinized tissue width (KT), gingival thickness (GT), pocket depth (PD), clinical attachment loss (CAL), pink esthetic score (PES), approximal plaque index (API), mean root coverage (MRC), and complete root coverage (CRC)—were assessed at baseline (T0), 3 months (T1), and 6 months (T2). **Results**: Both LDEE and DEE groups showed significant improvements in RD, KT, GT, PD, and CAL over time (*p* < 0.001). At T1 and T2, KT was significantly higher in the LDEE group (T1: 3.73 ± 0.72 mm; T2: 3.98 ± 0.76 mm) compared to the DEE group (T1: 3.21 ± 0.61 mm; T2: 3.44 ± 0.74 mm; *p* < 0.05). Other parameters (RD, GT, PD, CAL) showed no statistically significant intergroup differences at any time point (*p* > 0.05). After 6 months, MRC was 95% and CRC 82.6% for LDEE, compared to 94.8% and 82.6% for DEE (*p* > 0.05). PES scores were similar between groups at all time points (*p* > 0.05). **Conclusions**: Both laser- and scalpel-deepithelialized grafts effectively treated gingival recessions. LDEE combined with TUN resulted in significantly greater KT width compared to DEE + TUN.

## 1. Introduction

Gingival recession (GR), defined as the apical displacement of the gingival margin leading to root surface exposure, is a common clinical finding with multifactorial etiology. It arises from a combination of anatomical predispositions (e.g., thin gingival biotype, tooth malposition, and alveolar bone dehiscence), mechanical trauma (notably from improper brushing techniques), and chronic inflammation associated with dental biofilm [1,2,3,4,5,6,7]. GR becomes more prevalent with age and is reported more frequently in male patients [2]. Clinically, it may result in dentin hypersensitivity, compromised aesthetics, and increased susceptibility to root caries, all of which can significantly affect oral health-related quality of life. Management of GR requires a comprehensive approach involving behavioral modifications, plaque control, and—when indicated—periodontal plastic surgery to restore the gingival margin and increase soft tissue volume [4,7,8,9,10,11,12,13,14,15,16,17,18].

Microsurgical techniques have gained popularity due to their precision and tissue preservation, particularly in the context of minimally invasive interventions. Among these, erbium family lasers (Er:YAG, Er,Cr:YSGG) have shown promise due to their affinity for water-rich soft tissues, allowing for controlled ablation with minimal thermal damage and enhanced healing outcomes [19,20,21,22,23,24,25]. Historically, free gingival grafts (FGGs) were the first approach used for recession coverage but were limited by suboptimal aesthetics and discomfort at the donor site [26,27,28,29,30,31,32]. The introduction of subepithelial connective tissue grafts (SCTGs) significantly improved clinical results, especially in Miller Class I and II recessions, achieving over 90% root coverage in favorable cases [6,33,34,35,36]. These outcomes were further optimized by the use of coronally advanced flaps (CAF) [37], supraperiosteal envelope techniques (SET) [38], and tunnel techniques (TUN) [39,40], with TUN now favored for its minimal invasiveness, enhanced vascularization, and better integration of grafts [41,42,43].

Palatal connective tissue remains the gold standard for grafting, typically harvested between the canine and first molar while avoiding neurovascular structures [44,45,46]. Two primary harvesting strategies are recognized: (1) SCTG obtained via trap-door or single-incision under a partial-thickness flap, and (2) deepithelialized gingival grafts (DGG), derived from full-thickness grafts with subsequent epithelial removal using scalpels, burs, or lasers [47,48,49,50,51,52]. Among these, the Zucchelli technique is the most widely adopted for scalpel-based deepithelialization [25,50,52]. Compared to SCTG, DGGs are histologically denser and contain less glandular or adipose tissue, which may enhance their volume stability [44,53]. While acellular dermal matrix allografts (ADMAs) offer a donor-site-free alternative, autogenous grafts remain superior in keratinized tissue gain and healing dynamics [54,55,56].

The present study aimed to evaluate the clinical efficacy of laser-assisted deepithelialization of free gingival grafts using an Er,Cr:YSGG laser in the treatment of multiple gingival recessions. Specifically, the study assessed complete root coverage (CRC), mean root coverage (MRC), and changes in clinical parameters such as recession depth (RD), keratinized tissue width (KT), gingival thickness (GT), probing depth (PD), and clinical attachment level (CAL) at 3- and 6-month follow-ups. In addition, gingival aesthetics were evaluated using the Pink Esthetic Score (PES), with results compared between laser- and scalpel-deepithelialized grafts.

## 2. Materials and Methods

### 2.1. Study Design

**Figure 1:**
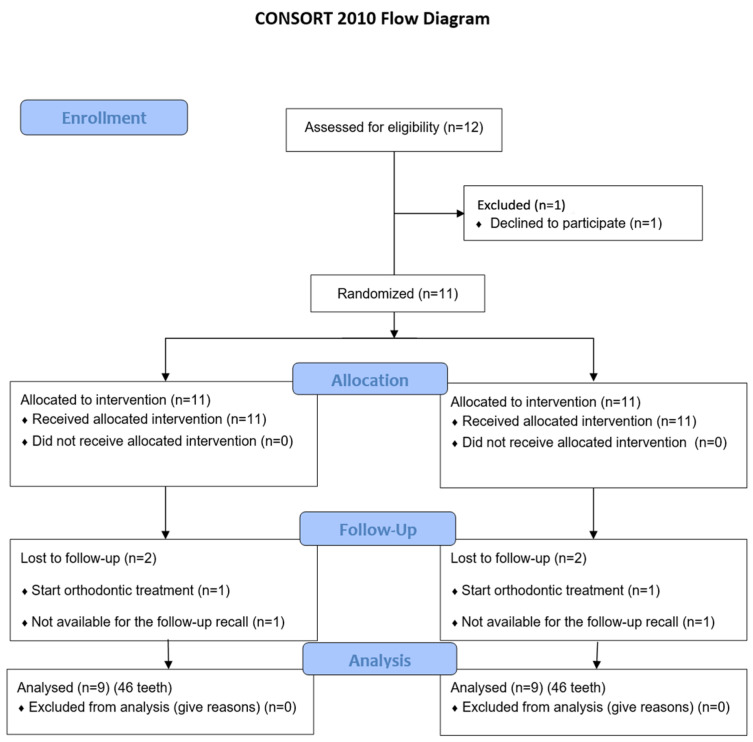
Flowchart of treated subjects according to CONSORT 2010.

This study was a single-center, single-blinded, split-mouth randomized clinical trial (RCT) structured according to the CONSORT statement (Figure 1). It evaluated the use of laser deepithelialization of the free gingival graft to cover gingival recessions. In the study group, the connective tissue graft (SCTG) was harvested by deepithelializing the free gingival graft using the Er,Cr:YSGG laser (LDGG), while in the control group, the traditional deepithelialization technique with a 15C scalpel (DGG) was used. The prepared grafts were then used to cover gingival recessions using the tunnel technique (TUN). A sample of palatal mucosa subjected to laser treatment was stained with hematoxylin and eosin (H&E) and examined histopathologically under 40× magnification.

A sample of palatal mucosa subjected to Er,Cr:YSGG laser deepithelialization was fixed in 10% buffered formalin immediately after harvesting. Following fixation, the tissue was dehydrated, embedded in paraffin, sectioned at 5 µm, and stained with hematoxylin and eosin (H&E). Histological evaluation was performed under 40× magnification using a light microscope (Leica DM500, Leica Microsystems, Wetzlar, Germany). The aim was to assess the depth and uniformity of epithelial removal, the integrity of the connective tissue, and the presence of thermal damage. A black ink marker was used to indicate tissue orientation. The analysis confirmed clear epithelial removal without signs of carbonization, coagulation, or connective tissue necrosis.

### 2.2. Sample Size

The study included 46 teeth with gingival recession (23 in the LDGG group and 23 in the DEE group), treated in 9 participants—2 men and 7 women—aged 19 to 51 years (mean age: 36.1 ± 8.46 years). The study group included 2 lateral incisors (8.7%), 9 canines (39.1%), 9 first premolars (39.1%), and 3 s premolars (13%). The control group included 1 lateral incisor (4.3%), 7 canines (30.4%), 9 first premolars (39.1%), and 6 s premolars (26.1%). All patients were treated between February 2021 and August 2022. The procedures were performed in full accordance with the principles of the Declaration of Helsinki (1975, revised in 2013), and the study design was approved by the District Medical Chamber in Bielsko-Biała (approval no. 2021/1/28/3). The trial was registered at the official website ClinicalTrials.gov (identifier: NCT07064304).

The sample size was calculated using G*Power software (Version 3.1.9.7, Kiel University, Kiel, Germany). Assuming an effect size (Cohen’s d) of 0.8 for the primary outcome (keratinized tissue width, KT), a two-tailed test, a significance level of α = 0.05, and a statistical power (1 − β) of 0.80, the minimum number of gingival recessions required per group was 21. Therefore, the total number of 46 treated recessions (23 per group) in the split-mouth design was deemed sufficient to detect clinically meaningful differences.

Patients were recruited through periodontal consultations at the dental center during the same period. Each eligible participant received comprehensive verbal and written explanations of the study objectives, procedures, and potential risks, and informed consent was obtained prior to inclusion. Participants were consecutively enrolled based on strict eligibility criteria, and no financial or therapeutic incentives were offered to avoid selection bias. The study was performed in Bielsko-Biala, where it received ethical approval and supervision from the District Medical Chamber (approval no. 2021/1/28/3), and was carried out as part of a master’s thesis within the European Master’s Degree in Oral Laser Applications (EMDOLA) at Wroclaw Medical University in Poland. The study complied with all ethical and methodological standards expected of university-based research and followed the Declaration of Helsinki.

All patients agreed to participate in the study and signed a written informed consent according to the above-mentioned principles. The patients were recruited based on the following inclusion criteria:

- age over 18;
- presence of at least two teeth with gingival recession RT1 [11] in the jaw in incisors, canines, premolars;
- buccal gingival recession defects ≥2–4 mm in depth;
- no periodontal inflammation (CPITN < 2);
- proper oral hygiene API < 15;
- probing depths < 3 mm;
- detectable cementoenamel junction (CEJ);
- no history of previous periodontal plastic surgery at the affected sites.

Exclusion criteria were as follows:

- smoker, also e-cigarettes;
- systemic diseases affecting periodontal status or healing (e.g., diabetes mellitus, immunodeficiency, osteoporosis, cardiovascular disease);
- intake of medications;
- presence of caries lesions or restorations in the cervical area;
- pregnancy and lactation.

### 2.3. Initial Therapy and Clinical Measurements

After the inclusion in this study, all patients were scheduled on a prophylaxis appointment during which they had scaling and professional tooth cleaning and were also instructed to use the roll technique in order to minimize mechanical trauma. Surgical treatment of the recession defects was not scheduled until the patient could demonstrate an adequate standard of supragingival plaque control. A single calibrated examiner, who was masked to the treatment allocation, performed all clinical measurements. Clinical measurements were recorded at the baseline, and at 3 and 6 months after surgeries.

**Figure 2:**
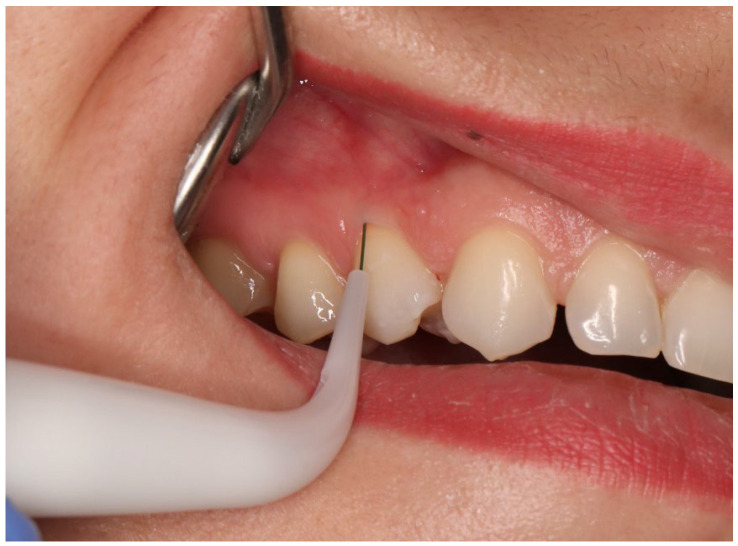
Electronic periodontal probe (PA-ON) examination.

The following parameters were assessed:

- recession depth (RD) measured at the mid-buccal aspect of the study tooth from the CEJ to the most apical extension of gingival margin using electronic periodontal probe PA-ON (Orangedental, Biberach, Germany); Figure 2;
- keratinized tissue (KT) measured from the most apical point of gingival margin to the muco-gingival junction (MGJ); the MGJ was identified by means of Lugol staining using a manual PCP-UNC 15 periodontal probe (Hu-Friedy, Chicago, IL, USA);
- gingival thickness (GT) measured at the mid-buccal aspect of the study tooth on a long axis, 2 mm apically from the gingival margin on CBCT slide (Kodak 8100 3D, Carestream/Trophy, Marne-la-Vallée, France), with a Field of View (FOV) of 5 cm × 4 cm, nominal beam of 73 kV, 12 mA and a voxel size of 150 µm. The measurement was valued by using Carestream 3D Suite (Carestream Health, Inc, Rochester, NY, USA);
- probing depth (PD) measured at the mesial, distal side, and mid-buccal aspect of the study tooth from the gingival margin to the bottom of the sulcus, PA-ON probe (Orangedental, Biberach, Germany); Figure 2;
- clinical attachment level (CAL) measured at the mid-buccal aspect of the study tooth from the CEJ to the bottom of the sulcus using electronic periodontal probe PA-ON (Orangedental, Biberach, Germany);
- approximal plaque index (API);
- community periodontal index of treatment needs (CPITN);
- mean root coverage (MRC), calculated as [(Baseline RD) − (6 months RD)/Baseline RD] × 100% [50];
- complete root coverage (CRC), calculated as the percentage of the teeth with recession defects having complete coverage achieved [(Teeth with CRC)/(All treated teeth)] × 100% [50];
- the graft area (GA), measured using the ImageJ (version 2.3.0/1.53t) Ver. G*Power (version 3.1.9.7) 1.53k computer program (https://imagej.nih.gov/ij accessed on 2 November 2024, NIH, Bethesda, MD, USA). Based on a 90-degree photo (Canon Eos 200, 135 mm + Yongnuo YN24EX flash ring) of the graft taken with a periodontal probe. Before the measurements, a calibration was performed, which consisted in marking a 15 mm reference section on periodontal probe PCP-UNC 15 (Hu-Friedy, Chicago, IL, USA);
- the pink aesthetics score (PES) developed by Furhauser et al. [57], assessed in the 3rd and 6th post-surgical month. It rates seven variables on a scale of 0 to 2 (mesial papilla, distal papilla, level of the gingival margin, marginal tissue contour, alveolar process contour, soft tissue color, soft tissue texture), yielding a maximum score of 14 points.

### 2.4. Randomization

Patients were assigned to one of two treatment groups using a computer-generated randomization table available at www.Randomizer.org (allocation ratio 1:1). The allocation was made secret through sealed, encoded opaque envelopes containing the treatment of a specific condition. The sealed envelope (containing treatment allocation) was opened during the procedure immediately before harvesting the graft.

### 2.5. Surgery

All surgical procedures were performed by the same oral surgeon in a microsurgical manner at 3× magnification (Heine, Germany). Immediately before the procedure, the root surfaces were cleaned with plastic brushes, and the contact points of the teeth were temporarily closed with a liquid, light-curing resin material (Filtek^®^ Supreme Flowable, 3M ESPE, St. Paul, MN, USA) to prevent the suspension sutures from collapsing. After administering local anesthesia (Septanest 1:200,000, Septodont, Saint-Maur-des-Fosses Cedex, France), the exposed root surfaces were prepared using Gracey curettes (Hu-Friedy, Chicago, IL, USA). A modified tunnel technique (TUN) was performed at all recipient sites [58]. Initial incisions in the gingival fissure were made with a microsurgical blade. The partial thickness tunnel was created with tunnel knives (14,446.02, 14,439.00 Stoma Dentalsysteme GmbH & Co KG, Emmingen-Liptingen, Germany) and extended beyond the MGJ to achieve mobilization and tension-free displacement of the flap towards the crown. FGG was obtained from the area between the distal line angle of the maxillary strip and the distal line angle of the first maxillary molar, sized according to the needs of the procedure. The full-thickness gingival graft was harvested from the palate using a 15c scalpel. Four incisions were made on the palate mucosa—two horizontal and two vertical- so that a rectangle was created that corresponded to the graft design. A coronal horizontal incision was made approximately 2 mm from the gingival margin. The graft was harvested using incisions parallel to the mucosa surface at an appropriate depth, leaving the periosteum undamaged. The graft was kept on sterile gauze dipped in saline where the graft was prepared. First, the adipose tissue and submucosa were removed from the periosteal side of the graft using a 15c scalpel. On the study side (LDEE), deepithelialization was performed using Er,Cr:YSGG, 2780 nm laser (Waterlase iPluse, Biolase, Foothill Ranch, CA, USA) with an MZ6 tip and the power of 2.5 W, 30 Hz, pulse duration: 60 µs, Water—80%, Air—70%, energy per pulse: 83.3 mJ, fluence (energy density per pulse) 28.6 J/cm^2^. During the procedure, the tip was positioned at an angle of 30–40 degrees to the graft surface and with a slow movement (1 mm/s) in light contact. Movements were made line by line across the entire width of the graft. The epithelium was deepithelialized twice in this way. Finally, the blunt end of the scalpel was used to scrape off the free tissue remnants after ablation. For the control side (DEE), deepithelialization was performed with a 15c scalpel [50]. The blade was set parallel to the graft surface, and horizontal incisions were made to a depth of about 0.5 mm. If necessary, the light was perpendicular during the procedure so that it was possible to better assess the remaining epithelium, which reflects the light better compared to connective tissue. After deepithelialization, the grafts were then placed in the tunnel with mattress sutures on the mesial and distal sides. Vertical double-crossed sutures (5-0 monofilament suture, Dogsan sutures, Trabzon, Turkey) were used to stabilize the cheek soft tissue complex 1–2 mm coronally to the CEJ (Zuhr et al.) [51]. In the case of covering three adjacent recessions, the graft was additionally individually stabilized to the adjacent tooth, and in the case of two recessions, no additional stabilization was used.

### 2.6. Post-Surgical Protocol

All patients were prescribed a soft diet, avoiding mechanical injuries, brushing, and flossing at the surgical sites for 2 post-surgical weeks until the suture was removed. To achieve plaque control, 0.12% Oral chlorhexidine gluconate (Eludril, Pierre Fabre, Paris, France) was used 3 times a day for 1 min for the first 2 weeks. Patients were prescribed 100 mg nimesulide (Nimesil, Laboratori Guidotti S.p.A, Pisa, Italy) and instructed to take one dose at the end of surgical intervention and one 12 h after surgery for anti-inflammatory and analgesic treatment. They were also instructed to take additional pain medication should it be needed. Two weeks after surgery, sutures were removed, and plaque control was maintained using an ultra-soft toothbrush (5/100) and a roller technique. One month after the sutures were removed, patients were allowed to return to normal oral hygiene procedures. All patients had a hygienic appointment for the improvement of oral hygiene at 2, 4, and 6 weeks and 3 and 6 months after the procedure.

### 2.7. Statistical Analyses

The teeth were divided into two groups: the control (DEE) and the studied group (LDGG). Linear Mixed–Effect Models (LMM) with Gaussian identity were applied. Because control and studied teeth occurred in the same patient, an effect of patient considered as random effect while effect of group (control vs. studied group) and time (baseline—T0, 3 months post-op—T3, 6 months post-po.—T6, delta T0–T3 and delta T0–T6) were treated as fixed factors in particular variables: RD, CAL, KT, GT, PD, PES. Also, the interaction of these factors was included. The Wald and chi-square statistics were calculated, as well as the *p*-value of significance of the model. In particular time series between the control (DEE) and studied (LDEE) groups, Student’s *t*-test was used. For area of transplant (TA), Student’s paired *t*-tests were used. The normality of distribution was checked by means of the Shapiro–Wilk test. All statistics and visualizations were calculated in R language and environment (version 4.2.1, R Core Team, 2022) using libraries Stats, lm4, car, and ggplot2.

## 3. Results

The study included a total of 46 gingival recessions in nine patients (seven women and two men), aged between 19 and 51 years (mean age 36.1 ± 8.46), all of whom completed the 6-month follow-up period (Table 1). Figure 1 shows the CONSORT flowchart diagram reporting the numbers of participants, who were randomly assigned, given intended treatments, and analyzed for the primary outcome of the present study. A total of 46 teeth were included in the study, 23 in the study group (LDGG) and 23 in the control group (DGG). In the study group, there were two lateral incisors (8.7%), nine canines (39.1%), nine first premolars (39.1%), and three second premolars (13%). The control group included one lateral incisor (4.3%), seven canines (30.4%), nine first premolars (39.1%), and six second premolars (26.1%) (Table 1). All patients had good oral hygiene before surgeries and throughout the follow-up period, maintaining API levels of 11.61 ± 1.76 at baseline, 11.95 ± 2.27 3 months post-op, and 12.2 ± 2.12 6 months post-op.

There were no statistically significant differences between the groups in all clinical parameters assessed at the baseline (Table 2).

### 3.1. Recession Depth (RD)

In the case of RD in the LDGG group at baseline, the mean values were 2.60 ± 0.72 mm; 3 months after the surgery, the mean values were 0.08 ± 0.28 mm; while after 6 months, they were 0.13 ± 0.30 mm. Accordingly, in the DGG group, the mean values were 2.83 ± 0.90 mm at baseline, 0.11 ± 0.29 mm after 3 months, and 0.15 ± 0.35 mm after 6 months. The results obtained in both groups showed statistical significance as a function of time at the level of *p* < 0.001. On the other hand, the RD values between the groups did not show statistical significance in any follow-up period. Similarly, there were no statistically significant values between the groups in the case of a change in RD values at the 3-month baseline (LDGG—2.52 ± 0.67 mm, DGG—2.72 ± 0.79 mm) and at the 6-month baseline (LDGG—2.39 ± 0.67 mm, DGG—2.68 ± 0.83 mm).

### 3.2. Probing Depth (PD)

For PD measurements in the LDGG group, the following results were achieved: 1.66 ± 0.34 mm, 1.38 ± 0.44 mm, and 1.47 ± 0.43 mm at baseline, after 3 months, and after 6 months, respectively. In the DGG group, the following results were obtained: 1.51 ± 0.36 mm at baseline, 1.18 ± 0.33 mm after 3 months, and 1.28 ± 0.40 mm after 6 months. The change in time △T0–T1 was 0.28 ± 0.33 mm for LDGG and 0.33 ± 0.41 mm for DGG, while for △ T0–T2, it was 0.32 ± 0.46 mm for LDGG and 0.23 ± 0.44 mm for DGG. Statistically significant results were obtained only for the function of time in both groups. Comparisons of the remaining results as a function of time did not show statistical significance.

### 3.3. Keratinized Tissue (KT)

In the case of KT, statistically significant values (*p* < 0.05) were obtained between the groups, both after 3 months (3.73 ± 0.72 mm—LDGG, 3.21 ± 0.61 mm—DGG) and after 6 months (3.98 ± 0.76 mm—LDGG, 3.44 ± 0.74 mm—DGG). The increase in KT also showed statistical significance between the groups at the level of *p* < 0.05 in favor of LDGG, both in the T0–T1 period (1.39 ± 1.16—LDGG and 1.09 ± 1.08—DGG, *p* < 0.05) and in the T1–T2 period (1.65 ± 1.19—LDGG and 1.43 ± 1.27—DGG, *p* < 0.05). In each group, the change in KT was also statistically significant (*p* < 0.001) as a function of time (Table 3).

### 3.4. Gingival Thickness (GT)

For GT in the LDGG group, the results were 0.83 ± 0.17 at baseline, 2.17 ± 0.31 after 3 months, and 2.09 ± 0.28 after 6 months. The increase in GT in the LDGG group showed statistical significance at the level of *p* < 0.001. Similar statistical significance was obtained in the DGG group, where the results were as follows: at baseline—0.86 ± 0.28, after 3 months—2.19 ± 0.41, and after 6 months—1.98 ± 0.35. Although in the LDGG group, higher values were obtained both after 3 and 6 months in relation to the DGG group, the difference was not statistically significant. The comparison of the increase between the groups also showed no statistically significant values.

### 3.5. Clinical Attachment Level (CAL)

CAL values for the LDGG group were 4.26 ± 0.66 at baseline, 1.12 ± 0.47 after 3 months, and 1.24 ± 0.58 after 6 months. For the DGG group, they were 4.34 ± 0.91 at baseline, 1.04 ± 0.65 after 3 months, and 1.15 ± 0.76 after 6 months. In both groups, the reduction in CAL showed statistical significance (*p* < 0.001) over the 6-month follow-up period. The reduction in CAL after 3 months was 3.47 ± 0.7 for the LDGG group and 3.65 ± 0.92 for the DGG group, respectively, and showed no statistical significance between the groups (Table 4).

### 3.6. Mean (MRC) and Complete (CMC) Root Coverage

The mean root coverage (MRC) measured 6 months after surgery was 95% in the LDGG group and 94.8% in the DGG group. Complete root coverage (CRC) was achieved in 19 of 23 (82.6%) gingival defects treated in both the study and control groups. The graft area in the LDEE group was 103.88 ± 18.61 mm^2^, while in the DGG group, it was 103.45 ± 14.02 mm^2^, and the values did not show statistical significance between the groups.

### 3.7. Pink Aesthetic Score (PES)

The pink aesthetic score (PES) was assessed 3 and 6 months after surgery. In the LDGG group, the values were 12.5 ± 0.73 (after 3 months) and 13.2 ± 0.8 (after 6 months) out of a possible 14 points. In the DGG group, the results were 12.5 ± 0.9 and 13.3 ± 0.64 3 and 6 months after surgery, respectively.

### 3.8. Graft Area (GA)

The mean values obtained for the graft area were very similar: 103.88 ± 18.61 mm^2^ for the LDGG group and 103.45 ± 14.02 mm^2^ for the DEE group. The results showed no statistically significant difference between the groups.

### 3.9. Histopathological Analysis

**Figure 3:**
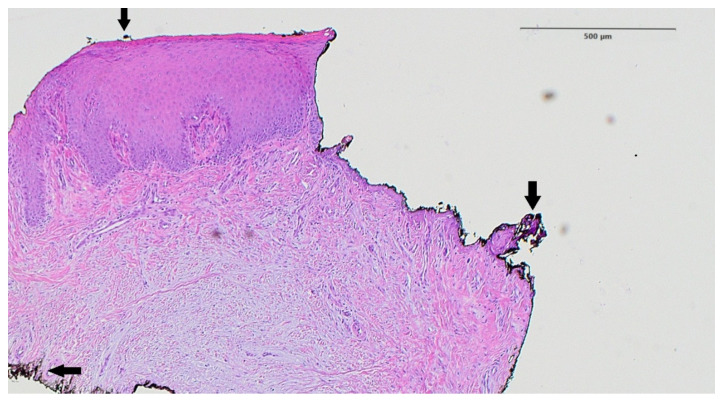
Histological analysis of the palatal mucosa after deepithelialization with the Er,Cr:YSGG laser according to the methodology proposed in this study. The border between the deepithelialized and the full-thickness part is shown. Arrow—black artifact caused by ink used to mark the specimen (hematoxylin–eosin staining, magnification ×40).

The histological slide clearly demonstrated a distinct boundary between the deepithelialized connective tissue and the remaining full-thickness epithelium, confirming controlled and uniform epithelial removal. No signs of carbonization, thermal necrosis, or structural damage to the underlying connective tissue were observed, suggesting that the Er,Cr:YSGG laser preserves tissue viability while ensuring effective epithelial ablation. A black ink artifact was visible in the slide, used for specimen orientation, but did not obscure diagnostic interpretation (Figure 3).

## 4. Discussion

The aim of this study was to evaluate the clinical application of gingival grafts harvested from the palate after deepithelialization using an Er,Cr:YSGG laser (2780 nm), compared to gingival grafts deepithelialized using the traditional scalpel technique. The study was designed as a split-mouth randomized clinical trial (RCT). The study focused on the incisors, canines, and premolars of the maxilla due to the similar anatomical characteristics of the soft tissues and tooth roots in this region. A total of 46 gingival recessions in nine patients were treated using either laser-deepithelialized grafts (LDGG) or scalpel-deepithelialized grafts (DGG). After 6 months, both groups achieved comparable mean root coverage (MRC: 95% in LDGG vs. 94.8% in DGG) and complete root coverage (CRC: 82.6% in both). The LDGG group showed significantly greater keratinized tissue width at 3 and 6 months (*p* < 0.05), while other clinical parameters (RD, PD, CAL, GT) improved significantly over time in both groups without intergroup differences. Aesthetic outcomes (PES) and graft areas were similar. Currently, lasers are utilized in dentistry as an alternative to conventional surgical instruments, and they have major advantages such as the production of local homeostasis [59,60], reduced postoperative pain and edema [57], bacterial elimination [61,62], the possibility of contact-free incision [63], and the avoidance of the need for sutures [59]. However, the use of the scalpel in oral surgery has inherent problems, such as difficulty in the visualization of the surgical field due to hemorrhage and excessive blood loss [59,64], while the bur can cause thermal necrosis and particle release to the atmosphere [63]. In the above study, the Er,Cr:YSGG laser with a wavelength of 2.78 μm was used, which is very well suited for working on soft tissues due to effective water absorption [63,65,66]. Investigations have shown that this type of laser may provide straight, clean, and precise ablation and cause minimal thermal damage to the adjacent tissue [66,67].

The safety and effectiveness of erbium lasers when working on soft tissues is confirmed, among others, by Monteiro et al. [68], who showed that erbium lasers cause the least thermal damage to tissues in surgical margins, compared to CO_2_, diode, Nd:YAG, and electroscalpels. Similar observations were made by Grzech-Leśniak [69], who showed the effectiveness of deepithelialization using the Er:YAG laser without thermal damage to the graft on animal material.

There are only a few reports in the literature on the use of the Er,Cr:YSGG laser for the deepithelialization of gingival graft. One of them is the study conducted by Lin et al. [70], who in their publication describe examples of the use of gingival grafts deepithelialized by Er,Cr:YSGG (2780 nm) laser. LDGGs have been used to cover recession, cover soft tissue fenestration at the implant, and provide soft tissue augmentation at the alveolar process. All treatments were successful, and a satisfactory clinical effect was obtained. Moreover, the authors insisted that it is easier to remove epithelium completely from FGG using an Er,Cr:YSGG laser than using a 15 scalpel, although the complete removal of epithelialization is clear in their histological evidence. Another publication written by Fekrezad et al. [71] describes the use of the Er,Cr:YSGG laser for deepithelialization of the gingival graft used to cover gingival recessions. They state that due to high superficial absorption, low penetration into the tissue, good incision, and safety for bones, the Er,Cr:YSGG laser may be useful at every stage of the free gingival graft transplant procedure. Gursoy [25] carried out a randomized clinical trial that evaluated gingival grafts deepithelialized using an Er:YAG laser to cover gingival recessions. He achieved statistically significantly greater thickness of the keratinized gingiva (GT) and better aesthetics compared to the control group where DGG by a scalpel was used. These results are consistent with our observations, where a higher GT was achieved in the LDGG group, but the results were not statistically significant. Among lasers, erbium lasers seem to be the best choice for deepithelialization of gingival grafts, while in the literature, there are also articles describing the use of other lasers such as diode and CO_2_ lasers. Although they show greater thermal damage to tissues, satisfactory clinical effects can also be obtained. According to Kawamura et. al. [72], they assessed the mean thermal damage caused by the CO_2_ laser at 236.6 µm and the 808 nm diode laser at 261 µm. These results are much higher compared to the Er:YAG and Er,Cr:YSGG lasers, where the results were 17.9 µm and 37.7 µm, respectively.

An example of the use of a diode laser for deepithelialization is the randomized clinical trial where Ozcelik et al. [73] performed deepithelialization using an 810 nm diode laser and assessed the use of LDGG in covering recessions. They obtained very good clinical results comparable to the control group, where the gingival graft deepithelialization by a scalpel was applied. On the other hand, Yoshino [74] describes the use of gingival grafts deepithelialized using a CO_2_ laser (10,600 nm) to cover gingival recessions. He found that this method could obtain good-quality LDGGs and increased the width of the keratinized gingiva by 2.9 ± 0.3 mm after 12-month follow-ups.

Many techniques for obtaining connective tissue have been proposed and applied. Among them, trapdoor and single incision techniques for harvesting SCTG are now widely used. In these conventional techniques, the thickness of the connective tissue is determined by the morphology of the palatal soft tissues. In many clinical situations, it is not possible to obtain a connective tissue graft of adequate quality if the soft tissue of the palate is not thick enough to harvest the connective tissue, and the techniques are not recommended due to the risk of necrosis of the primary flap and/or a suboptimal composition of the graft, which may include glandular and adipose tissue instead of connective tissue [50,75]. According to Harris [53], a thickness of the palate mucosa exceeding or equal to 3 mm guarantees the histologically correct quality of the graft. With a thin palate mucosa, it is recommended to harvest FGG, from which the epithelium is then removed. The DGG technique requires a smaller thickness of the palate mucosa to obtain the appropriate quality of the graft and to leave the appropriate thickness of the soft tissue covering the bone [50].

Bertl et al. [44] investigated the composition of gingival grafts, assessing fibrous, adipose, and glandular tissue using various techniques to harvest grafts on fresh human cadavers. They concluded that CTG obtained from deepithelialized gingival grafts (DGG) contained significantly larger amounts of connective tissue and significantly smaller amounts of adipose/glandular tissue than CTG obtained using the split-flap method. Another RCT [50] compared the results for root coverage with the use of CTG obtained using the trapdoor method and CTG obtained by removing the epithelium from FGG by scalpel. There were no significant differences in root coverage or KT growth between the two types of CTG harvesting techniques. On the other hand, there was a statistically significant difference in gingival thickness in 12-month follow-ups in favor of DGG, explained by the fact that DGG could have more dense, fibrous connective tissue that is more stable and has less tendency to disappear. Additionally, Bakhishov in his research showed a statistically significant increase in CRC and MRC in the group with DGG compared to SCTG in 1-year follow-ups [76].

The above analyses indicate that, thanks to DGG, compared to SCGG, it is possible to introduce into the graft a part of connective tissue closer to the epithelium, which is denser, firmer, more stable, and probably more suitable for root covering [53]. However, if we want to take a step forward and develop the technique of deepithelialization of the gingival graft, clinical evaluation of new technologies will be necessary. Laser deepithelialization of gingival grafts is a relatively new method and does not yet have an adequate scientific base. Therefore, this randomized clinical trial was conducted to compare DEE and LDEE in covering gingival recessions. In 6-month follow-ups, similar results were obtained in terms of MCR and CRC, which proves that LDGG is as effective in covering gingival recessions as DGG with a scalpel, which so far has been considered to be the gold standard. Moreover, in the study group, a statistically significant higher value of KT was obtained compared to the control group, both during the 3- and 6-month follow-ups and by analyzing the increase in KT after 3 months and after 6 months. A higher value of GT was also observed in the LDGG group in the 3- and 6-month follow-ups, although this result was not statistically significant. Promising results were obtained, which may indicate that the use of the Er,Cr:YSGG laser will allow for a controlled and even more precise removal of the epithelium, leaving the most desirable part of the connective tissue located just under the epithelium [53]. Perhaps thanks to this, it will be possible to obtain better and more predictable clinical results.

Moreover, as we know, procedures in the field of periodontal plastic surgery require exceptional surgical skills and extensive clinical experience. Surgeon-dependent factors significantly influence outcomes and should not be underestimated [77,78,79]. Deepithelialization using a scalpel is a technically demanding procedure, especially in the hands of an inexperienced surgeon. Therefore, utilizing a laser as an easier method to learn, with reproducible and constant physical parameters, enables predictable outcomes and reduces surgeon-dependent errors [80,81,82,83,84,85]. In addition to surgical outcomes, it is important to consider patient-centered factors when evaluating the clinical value of new technologies. Although the present study did not collect data on postoperative discomfort, pain, or healing experience, previous investigations have shown that laser-assisted procedures are associated with reduced postoperative morbidity due to minimized thermal and mechanical trauma. Therefore, future clinical trials should include validated patient-reported outcome measures (PROMs) to better evaluate the subjective aspects of healing and overall patient satisfaction [86,87,88,89]. From a practical standpoint, the adoption of Er,Cr:YSGG lasers in periodontal procedures also requires evaluation in terms of cost-effectiveness, equipment accessibility, and clinician learning curve. The high initial investment, need for training in laser physics and safety, and variation in technique-specific outcomes could present barriers in general and specialist practices. However, the reproducibility of laser parameters, the minimally invasive nature of the procedure, and the potential for improved patient comfort and reduced complications may offset these challenges in selected cases—especially when graft precision and soft tissue preservation are critical to the outcome.

In this study, the pink aesthetic score (PES), according to Furhanser [57], was used to assess aesthetics. It was initially developed to evaluate the aesthetics of implants but was adopted by Zuhr and Hulzeler [49,51] to evaluate aesthetics following periodontal procedures and is particularly suitable for this purpose. This indicator is less commonly used than the method described by Cairo [79]; however, it offers a more comprehensive evaluation of soft tissue parameters, as highlighted in this study. The aesthetic effects obtained in both groups were comparable and considered excellent. Similarly, Gursoy et al. [25] achieved very good aesthetic results in recession coverage using LDGG harvested with an Er:YAG laser, which were even better compared to the traditional deepithelialized gingival graft (DGG) performed with a scalpel.

## 5. Conclusions

Within the limitations of this study, it can be concluded that both gingival grafts deepithelialized using an Er,Cr:YSGG laser and those deepithelialized with a scalpel effectively achieve coverage of gingival recessions. The clinical outcomes, expressed as mean root coverage (MRC) and complete root coverage (CRC), were comparable between both treatment groups. Notably, the use of laser-deepithelialized gingival grafts (LDGG) combined with the tunnel technique (TUN) resulted in greater keratinized tissue (KT) width compared to the scalpel-deepithelialized gingival graft (DGG) combined with TUN. Both approaches also demonstrated similarly favorable aesthetic outcomes at the 6-month follow-up evaluation. These preliminary findings suggest that laser-assisted deepithelialization may be a predictable and reproducible alternative to conventional techniques, particularly in cases where soft tissue precision and preservation are critical. However, further randomized clinical trials involving larger patient populations are needed to confirm these results. Future studies should also include patient-reported outcome measures (PROMs) and assess the feasibility of laser implementation in routine dental practice, considering factors such as cost-effectiveness, training requirements, and overall patient experience.

## Figures and Tables

**Table 1:** Demographic data and the characteristics of study subjects.

	LDGG	DGG
Patient	9 (100%)	
Males	2 (2.2%)	
Females	7 (77.7%)	
Age (range)	36.1 ± 8.46 (19–51)	
Total teeth	23 (100%)	23 (100%)
Mesial incisors	0 (0%)	0 (0%)
Lateral incisors	2 (8.7%)	1 (4,3%)
Canines	9 (39.1%)	7 (30.4%)
First premolar	9 (39.1%)	9 (39.1%)
Second premolar	3 (13%)	6 (26.1%)
API		
Baseline	11.61 ± 1.76	
3 months post-op.	11.95 ± 2.27	
6 months post-op	12.2 ± 2.12	
CPITN	1.44 ± 0.53	

LDGG—Laterally Displaced Gingival Graft; DGG—Double Gingival Graft; API—Approximal Plaque Index; CPITN—Community Periodontal Index of Treatment Needs.

**Table 2:** Clinical parameters (mean and SD) at baseline (T0), 3 months post-op. (T1) and 6 months post-op. (T2).

	Baseline (T0)	3 Months (T1)	6 Months (T2)	*p*-Value
RD				
LDEP	2.60 ± 0.72	0.08 ± 0.28	0.13 ± 0.30	*p* < 0.001
DEP	2.83 ± 0.90	0.11 ± 0.29	0.15 ± 0.35	*p* < 0.001
*p*-value	NS	NS	NS	
PD				
LDEP	1.66 ± 0.34	1.38 ± 0.44	1.47 ± 0.43	*p* < 0.001
DEP	1.51 ± 0.36	1.18 ± 0.33	1.28 ± 0.40	*p* < 0.001
*p*-value	NS	NS	NS	
KT				
LDEP	2.19 ± 1.18	3.73 ± 0,72	3.98 ± 0.76	*p* < 0.001
DEP	2.25 ± 0.71	3.21 ± 0.61	3.44 ± 0.74	*p* < 0.001
*p*-value	NS	*p* < 0.05	*p* < 0.05	
GT				
LDEP	0.83 ± 0.17	2.17 ± 0.31	2.09 ± 0.28	*p* < 0.001
DEP	0.86 ± 0.28	2.19 ± 0.41	1.98 ± 0.35	*p* < 0.001
*p*-value	NS	NS	NS	
CAL				
LDEP	4.26 ± 0.66	1.12 ± 0.47	1.24 ± 0.58	*p* < 0.001
DEP	4.34 ± 0.91	1.04 ± 0.65	1.15 ± 0.76	*p* < 0.001
*p*-value	NS	NS	NS	

**Table 3:** Change in clinical parameters at baseline—3 months post-op. (△T0–T1) and baseline—6 months post-op. (△T0–T2).

	△T0-T1	△T0-T2
RD		
LDEP	2.52 ± 0.67	2.47 ± 0.67
DEP	2.72 ± 0.79	2.68 ± 0.83
*p*-value	NS	NS
PD		
LDEP	0.28 ± 0.33	0.19 ± 0.36
DEP	0.33 ± 0.41	0.23 ± 0.44
*p*-value	NS	NS
KT		
LDEP	1.54 ± 1.16	1.79 ± 1.19
DEP	0.96 ± 1.08	1.19 ± 1.27
*p*-value	*p* < 0.05	*p* < 0.05
GT		
LDEP	1.34 ± 0.22	1.26 ± 0.22
DEP	1.33 ± 0.37	1.12 ± 0.33
*p*-value	NS	NS
CAL		
LDEP	3.14 ± 0.7	3.02 ± 0.66
DEP	3.3 ± 0.92	3.19 ± 0.99
*p*-value	NS	NS

**Table 4:** Values (mean and SD) of complete root coverage (CRC), mean root coverage (MRC), graft area (GA), and pink aesthetic score (PES) for LDGG and DGG site.

	MRC	CRC	GA [mm^2^] (Range)	PES	
	T2	T2	T0	T1	T2
LDEP	95%	82.6%	103.88 ± 18.61 (77.18–120.24)	12.5 ± 0.73	13.2 ± 0.8
DEP	94.8%	82.6%	103.45 ± 14.02 (75.5–127.31)	12.5 ± 0.9	13.3 ± 0.64
*p*-value	NS	NS	NS	NS	NS

## Data Availability

The original contributions presented in the study are included in the article, further inquiries can be directed to the corresponding authors.
